# Pushing the limits of magnetic anisotropy in trigonal bipyramidal Ni(ii)†
†Electronic supplementary information (ESI) available. CCDC 1059709. For ESI and crystallographic data in CIF or other electronic format see DOI: 10.1039/c5sc02854j



**DOI:** 10.1039/c5sc02854j

**Published:** 2015-09-08

**Authors:** Katie E. R. Marriott, Lakshmi Bhaskaran, Claire Wilson, Marisa Medarde, Stefan T. Ochsenbein, Stephen Hill, Mark Murrie

**Affiliations:** a WestCHEM , School of Chemistry , University of Glasgow , Glasgow , G12 8QQ , UK . Email: mark.murrie@glasgow.ac.uk; b Department of Physics and NHMFL , Florida State University , Tallahassee , FL 32310 , USA . Email: shill@magnet.fsu.edu; c Laboratory for Developments and Methods , Paul Scherrer Institute , CH-5232 Villigen PSI , Switzerland; d Laboratory for Neutron Scattering and Imaging , Paul Scherrer Institute , CH-5232 Villigen PSI , Switzerland

## Abstract

Monometallic complexes based on 3d transition metal ions in certain axial coordination environments can exhibit appreciably enhanced magnetic anisotropy, important for memory applications, due to stabilisation of an unquenched orbital moment. For high-spin trigonal bipyramidal Ni(ii), if competing structural distortions can be minimised, this may result in an axial anisotropy that is at least an order of magnitude stronger than found for orbitally non-degenerate octahedral complexes. Broadband, high-field EPR studies of [Ni(MDABCO)_2_Cl_3_]ClO_4_ (**1**) confirm an unprecedented axial magnetic anisotropy, which pushes the limits of the familiar spin-only description. Crucially, compared to complexes with multidentate ligands that encapsulate the metal ion, we see only a very small degree of axial symmetry breaking. **1** displays field-induced slow magnetic relaxation, which is rare for monometallic Ni(ii) complexes due to efficient spin–lattice and quantum tunnelling relaxation pathways.

## Abstract 2

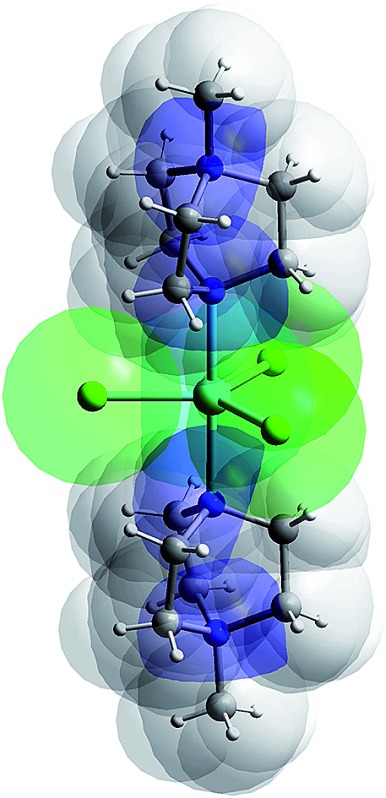
High-field EPR and magnetic studies of a high-spin Ni(ii) trigonal bipyramidal complex reveal a giant axial magnetic anisotropy and a rare field-induced slow magnetic relaxation.

## Introduction

Understanding and controlling magnetic anisotropy at the level of a single metal ion is vital if the miniaturisation of data storage is to continue to evolve into transformative technologies.^1^ IBM recently demonstrated that, at low temperature, nanoscale arrays of surface Fe atoms are potential candidates for magnetic memory and spintronics applications.^2^ Furthermore, by coordinating a single Co atom to the O site of an MgO (100) surface, a giant uniaxial magnetic anisotropy of 57.7 meV (∼465 cm^–1^) was reported.^3^ The role of axial magnetic anisotropy is to pin the magnetic moment of the metal ion in one of two preferred orientations, either parallel or antiparallel to the magnetic easy-axis. For transition metals, maximisation of the axial magnetic anisotropy requires stabilisation of an unquenched orbital moment that can couple to an axial ligand field. Importantly, these parameters can be designed *in silico* for monometallic 3d transition metal complexes.^4^ This prior engineering of the magnetic anisotropy, based upon coordination number and electronic structure, can then be realised using a bottom-up synthetic approach.

Multinuclear complexes based on the controlled assembly of a small number of ions with such optimised magnetic anisotropy are key targets for increasing single-molecule magnet (SMM) blocking temperatures. The first step is to design the potential building blocks in order to maximise the magnetic anisotropy, but for axial monometallic complexes, the zero-field splitting |*D*| is commonly much less than 100 cm^–1^.^5^ However, it should be possible to chemically engineer this anisotropy to approach the theoretical limit determined purely by the one electron spin–orbit coupling parameter [668 cm^–1^ for a Ni(ii) free ion].^6^ For high-spin trigonal bipyramidal (TBP) Ni(ii), the axial anisotropy should be at least an order of magnitude stronger than found for octahedral (|*D*| ≈ 10 cm^–1^)^7^ or square pyramidal complexes (|*D*| ≈ 15 cm^–1^),^8^ due to an orbitally degenerate ground state.^9^

**Fig. 1:**
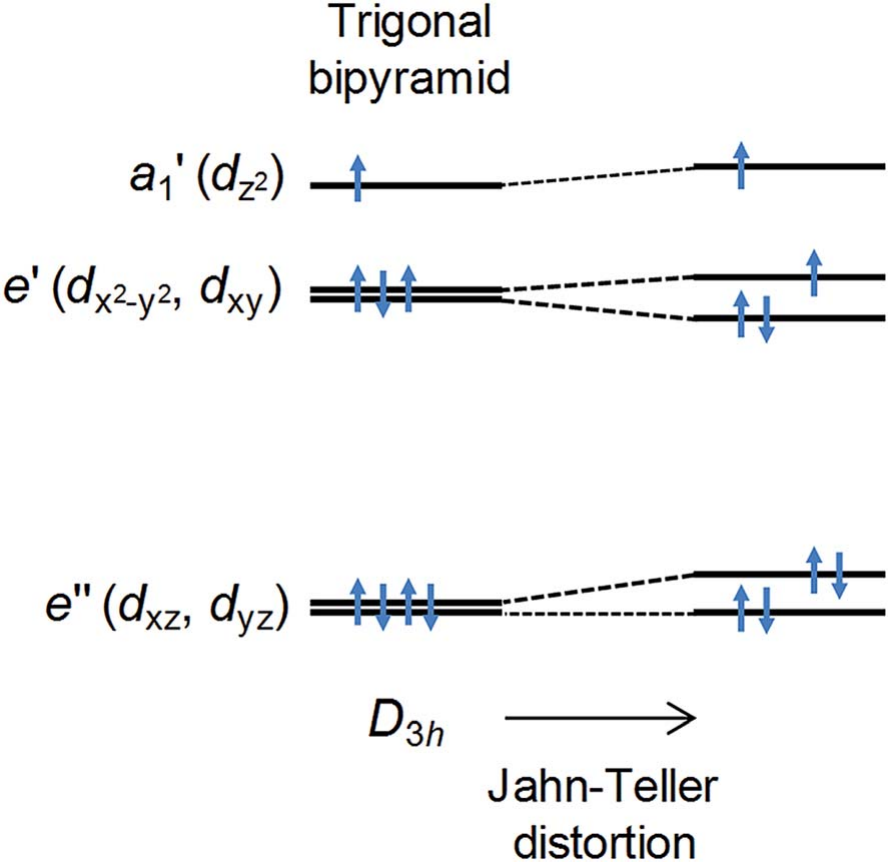
d-orbital splitting for high-spin Ni(ii) in an ideal trigonal bipyramidal environment (left) and the effect of a symmetry-lowering Jahn–Teller distortion that removes the orbital degeneracy (right).

In the ideal high-spin TBP Ni(ii) (d^8^) case, three electrons reside in the degenerate d_*xy*_ and d_*x*^2^–*y*^2^_ orbitals (Fig. 1), leading to an unquenched orbital moment and the desired giant first order contribution to the spin–orbit coupling (SOC) anisotropy. However, this orbitally degenerate state is typically unstable with respect to Jahn–Teller distortion (Fig. 1) away from the ideal trigonal geometry, leading to (i) a quenching of the first order SOC and an overall reduction in the axial anisotropy, and (ii) the generation of non-axial SOC terms that mix spin-up and -down states, giving rise to magnetic quantum tunnelling effects that are antagonistic to SMM behaviour. It has been predicted using computational methods that minimising these unwanted structural distortions, which involve deformations of the equilateral triangle formed by the equatorial ligands and/or a bending of the axial ligand–metal bonds away from 180°, should lead to an almost unquenched orbital moment.^10^ This can be done by combining large ligands in the equatorial positions along with rigid, bulky ligands in the axial positions. We now report broadband, high-field EPR studies of [Ni(MDABCO)_2_Cl_3_]ClO_4_ (**1**) (MDABCO^+^ = 1-methyl-4-aza-1-azoniabicyclo[2.2.2]octanium cation) that show an unprecedented magnetic anisotropy, reaching the limits of applicability of the familiar spin-only description: a *D* parameter in the range from –400 to –535 cm^–1^ is estimated on the basis of such an analysis, the largest found so far for a Ni(ii) complex; and, importantly, only a very small degree of axial symmetry breaking can be detected, with an upper-bound for the *E* parameter of 0.18 cm^–1^ within this *S* = 1 description.

## Results and discussion

### Synthesis and structure

**Fig. 2:**
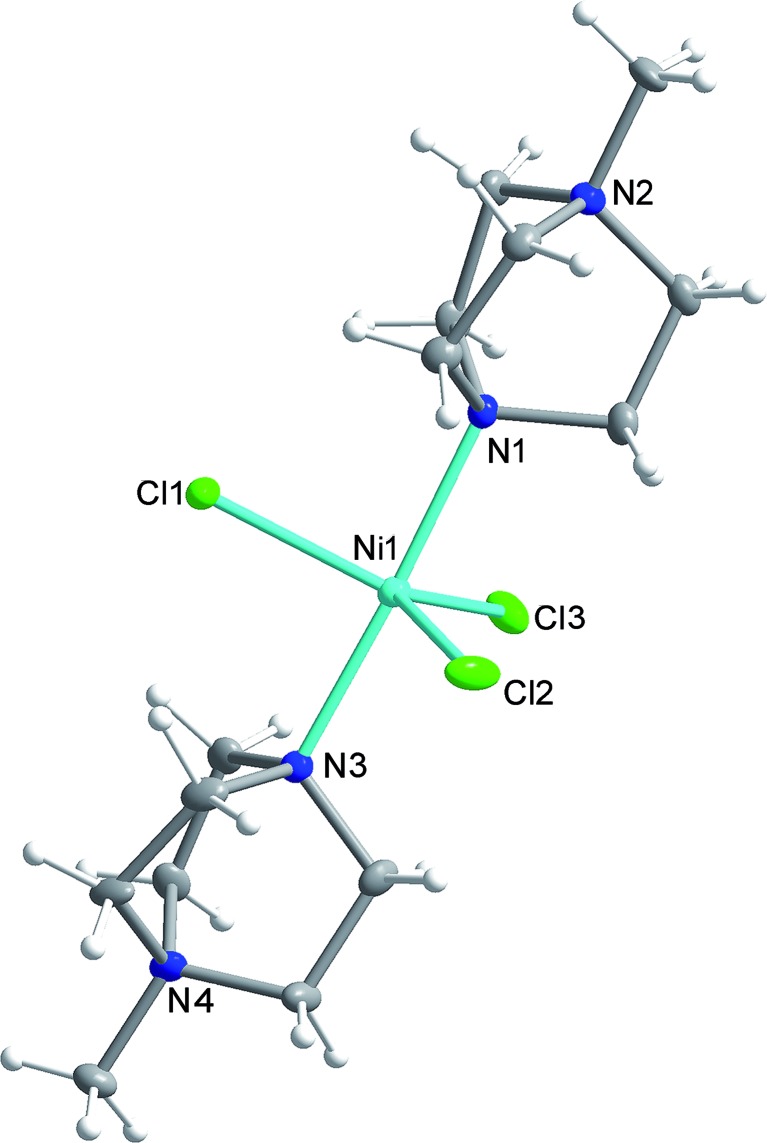
The structure of the [Ni(MDABCO)_2_Cl_3_]^+^ cation in **1** with ellipsoids drawn at 50% probability level. C, grey; Cl, green; H, white; N, blue; Ni, cyan.

Compound **1** was synthesised as described in the literature, with slight modification (see ESI†),^11,12^ and crystallises in the orthorhombic space group *Pca*2_1_ with one [Ni(MDABCO)_2_Cl_3_]ClO_4_ unit in the asymmetric unit (Fig. 2 and Table S1†) and four differently oriented molecules within the unit cell (Fig. S2†). The Ni(ii) centre exhibits trigonal bipyramidal geometry, with two [MDABCO]^+^ ligands occupying the axial positions and three chloride ligands in the equatorial positions (Fig. 2). There is a small distortion of the trigonal bipyramidal geometry around the Ni centre (Table S2†) [bond angles: Cl–Ni–N = 88.58–91.41°; Cl–Ni–Cl = 117.03–123.24°; N–Ni–N = 176.59 (19)]. Continuous shape measures,^13^ which provide an estimate of the distortion from the ideal trigonal bipyramidal structure, give a value of 0.133 (where 0 corresponds to the ideal polyhedron), confirming a small distortion, which is important in terms of the magnetic behaviour (*vide infra*). For comparison, in [Ni(Me_6_tren)(Cl)]ClO_4_ where the magnetic anisotropy is smaller, the distortion from TBP is much higher (35.256 *cf.* 0.133).^9^

### DC magnetic measurements

**Fig. 3:**
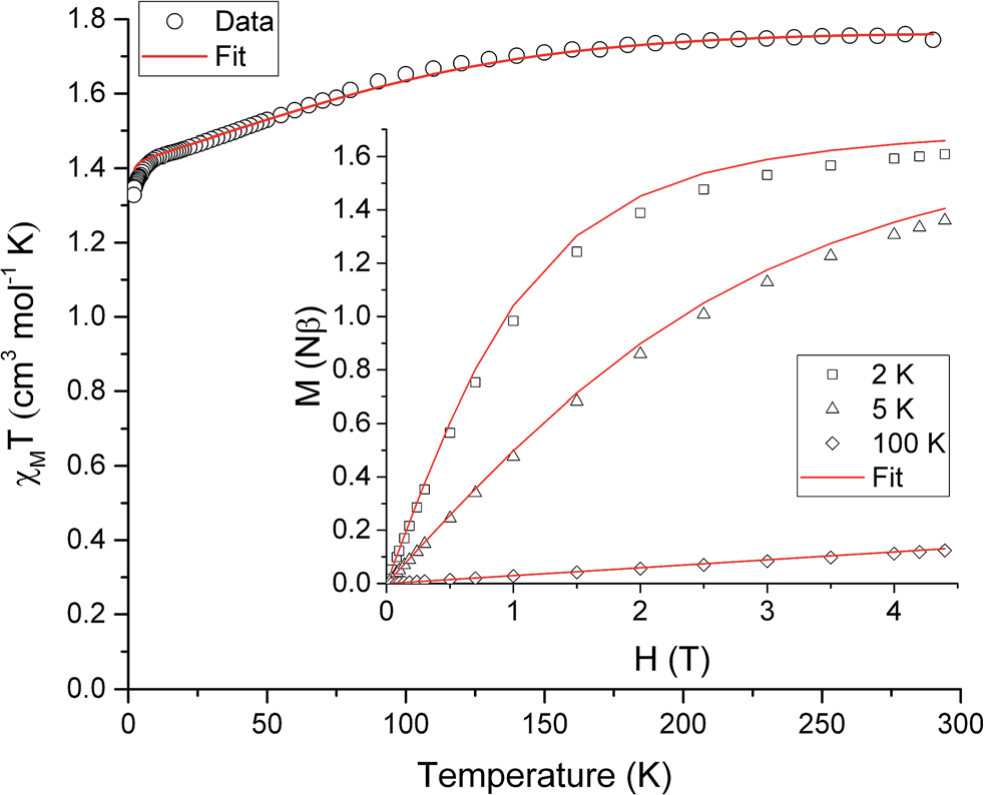
Variable temperature DC susceptibility data for **1** in a field of 1 kOe from 300–1.8 K. Inset: reduced magnetisation *versus* field at 2, 5 and 100 K. Solid lines represent the best simultaneous fit for the experimental data (see text for details).

The ambient temperature *χ*_M_ *T* value of 1.75 cm^3^ mol^–1^ K (Fig. 3) is consistent with the presence of a significant orbital contribution to the magnetic moment (for comparison, *χ*_M_ *T*_calc_ for Ni(ii) in an octahedral environment, where the orbital moment is largely quenched, is 1.16 cm^3^ mol^–1^ K (for *g* = 2.15)). The DC magnetic susceptibility measurements and magnetisation curves were fitted simultaneously using the program *Phi*,^14^ as described by the following effective spin Hamiltonian (eqn (1)):1
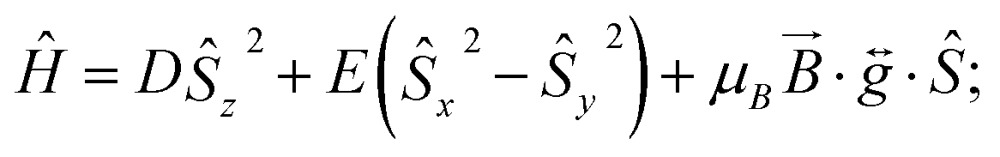
where the 1st and 2nd terms characterise the axial and rhombic anisotropic zero-field-splitting (zfs) interactions, parameterised by *D* and *E*, respectively; *ŝ* is the spin operator with components *ŝ*_*i*_ (*i* = *x*, *y*, *z*); and the final term denotes the Zeeman interaction with the local magnetic field, *B*, parameterised through the Landé 
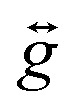
 tensor. Initially, within *Phi*, simulations were carried out to explore the parameter space and it became clear that anisotropic *g* values were required to model the *χ*_M_ *T vs. T* data in addition to the zfs parameters. In order to reduce the number of parameters, *g*_*z*_ = 3.36 and *E* = 0.18 cm^–1^ were taken from the EPR data (*vide infra*). Given the very weak rhombicity, the constraint *g*_*x*_ = *g*_*y*_ was also applied. Simultaneous fits of the susceptibility and magnetisation data (see Fig. 3) give *D* = –311 (20) cm^–1^, and *g*_*x*_ = *g*_*y*_ = 2.05 (2).^15^ Note that the large value of *g*_*z*_ is essential to obtain a good fit, indicating mixing of a considerable orbital moment into the ground state.

### High-field EPR

**Fig. 4:**
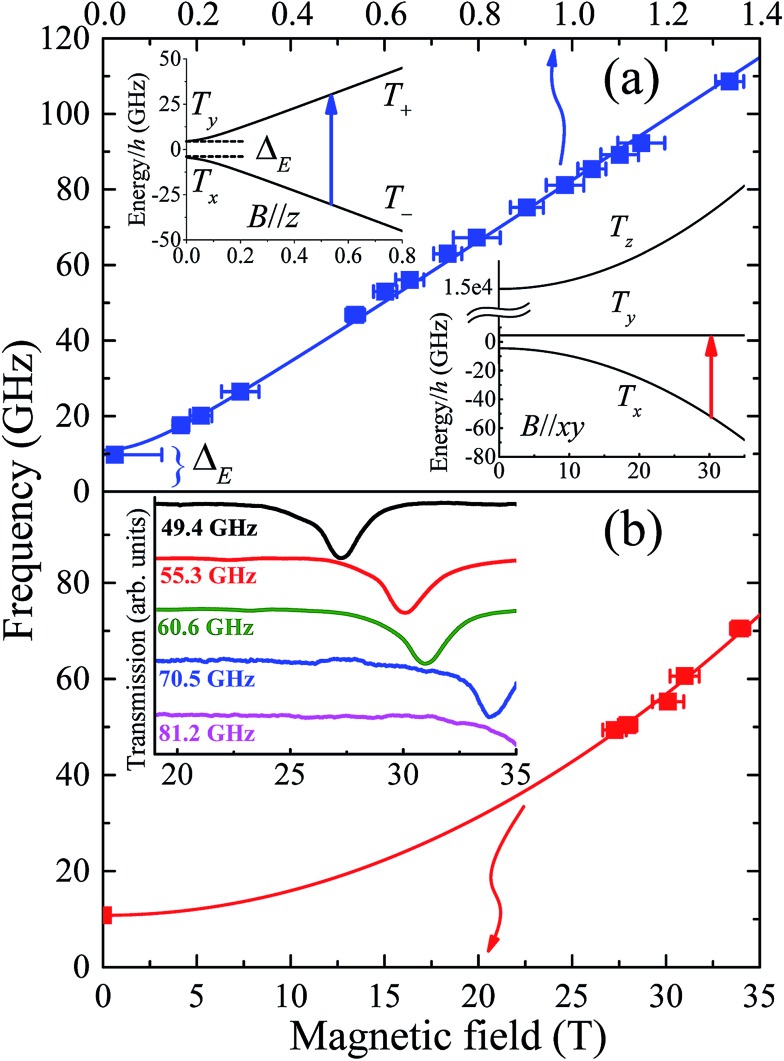
(a) Frequency dependence of low-field EPR peak positions associated with transitions between the lowest-lying pair of (pure) triplet states, T_+_ and T_–_ (see upper inset); the temperature was 4.2 K and the applied field estimated to be ∼30° away from the easy-(*z*-) axis for these measurements (see Fig. S4† and main text for further details). The lower inset in (a) depicts the energy level diagram appropriate to the situation in (b) which plots the high-field EPR peak positions associated with the same pair of levels, with the applied field now oriented exactly within the *xy*-plane of the molecule (the strongly admixed states are labelled T_*x*_ and T_*y*_ in this case, according to the low-field representation) and the temperature is 4.2 K. The inset to (b) displays actual high-field spectra, with the dips in transmission corresponding to resonances.

Assuming a spin-only description for a d^8^ ion such as Ni(ii), with a large Ising-type anisotropy, the (effective) triplet energy level diagram consists of a pair of low-lying singlets that are very well isolated from the third singlet state (see insets to Fig. 4a), as described by eqn (1). Direct measurement of the axial anisotropy parameter *D* would require excitation of EPR transitions from the low-lying singlets to the excited level, which is estimated to lie some 400 to 535 cm^–1^, or 12–16 THz above (*vide infra*) in the present case, rendering it inaccessible to essentially any currently available EPR spectrometer.^16^ However, recent studies have demonstrated that application of a large magnetic field transverse to the easy-(*z*-) axis enables an indirect estimation of *D* from the low-frequency transition between the lowest-lying singlets (see lower right inset to Fig. 4a).^9,16^ Meanwhile, these levels are themselves split in zero-field by the rhombic interaction (zero-field gap Δ*_E_* = 2*E*, see upper left inset and main panel of Fig. 4a).

Single-crystal EPR measurements provide significantly enhanced sensitivity relative to more widely employed powder techniques in cases where the spectrum spans an extremely wide magnetic field range,^16^ as is the case for highly anisotropic species such as **1**. However, the gain in sensitivity comes at the cost of introducing two additional parameters – the polar (*θ*) and azimuthal (*φ*) angles that define the orientation of the applied field relative to the local coordinates of the magnetic species under investigation. This complicates matters considerably in the case of compound **1** due to its low symmetry space group and the existence of four differently oriented molecules within the unit cell (Fig. S2†). Therefore, in order to maximally constrain the various spin Hamiltonian parameters, measurements were separately performed in several different low-field spectrometers, in addition to the angle-dependent high-field single-crystal measurements that primarily constrain the *D* parameter. We begin by summarising the low-field results.

Powder spectra were collected in the 50 to 225 GHz frequency range in order to accurately constrain the parallel component of the Landé tensor, *g*_*z*_. Representative derivative-mode spectra are displayed in the inset to Fig. S4† along with a plot of the resonance positions *versus* frequency in the main panel. Although there are four differently oriented molecules within the unit cell, they are related by symmetry. Consequently, the powder spectra for these four molecules are identical. The lowest field component of the powder spectrum corresponds to the parallel-mode, double-quantum transition (Δ*m*_S_ = 2) between the low-lying T_+_ and T_–_ singlets for molecules oriented with *B*//*z* [see upper-left inset to Fig. 4a]. The effective *g*-value, *g*_eff_ = 6.72 (6), deduced from this transition is twice the parallel component of the Landé tensor associated with the *S* = 1 effective spin multiplet, *i.e.*, *g*_*z*_ ≈ 3.36 (3). This value was thus used to constrain the fit (Fig. 3) to the magnetic data that, in turn, provides a more reliable estimate of the perpendicular component of the *g*-tensor.

As noted above, the zero-field gap between the T_+_ and T_–_ singlets provides a direct measure of *E*. However, neither the high-field or powder EPR spectrometers are capable of accessing frequencies below ∼50 GHz due to restricted magnet bore sizes, requiring the use of narrow waveguides (this is a fundamental limitation of high-field magnet systems). Consequently, a series of low-field single-crystal measurements were conducted in the 17 to 110 GHz range, using a horizontal split-pair magnet that allowed for rotation of the sample about a single axis. Spectra were first collected as a function of field orientation, revealing four independent Δ*m*_S_ = 2 resonances, *i.e.*, one for each of the different molecular orientations. The lowest-field peak, corresponding to closest alignment of the applied field with respect to the easy-axis of one of the four molecules (estimated to be ∼30° away from its *z*-axis on the basis of the powder data, see Fig. S4†), was then selected for frequency-dependent studies. These measurements were augmented by a single measurement at 9.7 GHz using a commercial X-band spectrometer, revealing a broad peak centred at zero field. The combined data (Fig. 4a main panel) suggest an upper bound on Δ*_E_* of ∼11 GHz (*E* ≤ 0.18 cm^–1^). This is a remarkably small value considering that the local coordination geometry around the Ni(ii) ion of compound **1** is not rigorously trigonal; it should be noted that the Ni(ii) complex reported in ref. 9, which possesses a trigonal structure, undergoes a Jahn–Teller distortion resulting in an *E* value (1.6 cm^–1^) that is an order of magnitude larger than found here. These measurements therefore suggest a greater structural rigidity in **1** compared to [Ni(Me_6_tren)(Cl)]ClO_4_ that prevents Jahn–Teller-type physics that could potentially reduce the first-order SOC contribution to the axial anisotropy.

To test the above hypothesis, very high-field measurements were performed with a view to determining the axial zero-field splitting parameter, *D*. Again, angle-dependent measurements were first performed (Fig. S5†). Due to the strong axial zero-field anisotropy, the lowest pair of singlets experience a linear Zeeman splitting with respect to the longitudinal field component (*B*//*z*) and a considerably weaker non-linear dependence on the transverse component [*B*//*xy*, see lower-right inset to Fig. 4a]. Consequently, as the field orientation approaches the *xy* plane associated with one of the four molecules in the unit cell, the corresponding ground state EPR transition between the low-lying T_*xy*_ singlets moves very rapidly to high fields (see Fig. S5†). In this way, one can locate the *xy*-plane for each molecule by carefully tracking the corresponding resonance to its highest field position. This field orientation is then selected for further frequency dependent studies (Fig. 4b). It should be noted that the angle-dependent measurements are extremely challenging, time consuming and costly, due to the strong anisotropy of compound **1** which results in narrow angle ranges where an EPR peak can move from 15 to 35 T in just 3 degrees of rotation (see Fig. S5†). The fact that sharp resonances can be observed in these regions is also a testament to the exceptionally high quality of the crystals, indicating very little orientational disorder.

As noted previously,^9^ the *D* parameter deduced from a fit to the frequency dependent data (Fig. 4b) is highly sensitive to *E*, as well as to *g*_*x*_ and *g*_*y*_. Moreover, precise knowledge of the plane of field rotation (particularly with respect to *x* and *y*) is essential if significant rhombicity is present. However, the low-field measurements indicate very weak rhombicity (*E* ≤ 0.18 cm^–1^), allowing us to set *g*_*x*_ = *g*_*y*_ = *g*_*xy*_. Meanwhile, low-field powder EPR measurements indicate *g*_*z*_ ≈ 3.36 (3) (see Fig. S4†), requiring *g*_*xy*_ ≈ 2.05 in order to account for the magnetic data. Based on these assumptions, the best fit to the frequency dependent high-field EPR data in Fig. 4b suggest *D* = –535 ± 5 cm^–1^, resulting in a corresponding zero-field gap of ≈535 cm^–1^ between the ground (T_*x*_ & T_*y*_) and excited (T_*z*_) triplet states. This gap is close to the theoretical maximum one would expect on the basis of an orbital description, as set by the one electron SOC parameter, *i.e.*, 668 cm^–1^ for the Ni(ii) free ion.^6^ Although the *E* parameter is small, its finite size and lack of knowledge of the field orientation within the *xy*-plane does contribute some softness in the *D* value quoted above, as do the uncertainties in *g*_*xy*_; a very conservative analysis gives a lower bound of about –400 cm^–1^ (see Fig. S6†). This analysis obviously pushes the limits of the spin-only model. However, there is no scenario in which the high-field EPR data can be explained with a much smaller absolute value of the *D* parameter (<400 cm^–1^), for which the spin-only description would be quite appropriate. For comparison, in [Ni(Me_6_tren)(Cl)]ClO_4_, the magnitude of the *D* parameter is estimated to be much lower, *i.e.*, less than 120 cm^–1^, as the Jahn–Teller distortion lifts the orbital degeneracy associated with the ground term, which reduces the first-order spin–orbit contribution to the axial magnetic anisotropy. A simple side-by-side comparison of Fig. 4b with the high-field EPR data reported for [Ni(Me_6_tren)(Cl)]ClO_4_ in ref. 9 confirms a much stronger axiality and overall anisotropy in the present case, *i.e.*, **1** possesses a much smaller zero-field gap (Δ_*E*_) between the low-lying pair of singlets, and a far weaker field dependence of this gap.

### AC magnetic susceptibility measurements

**Fig. 5:**
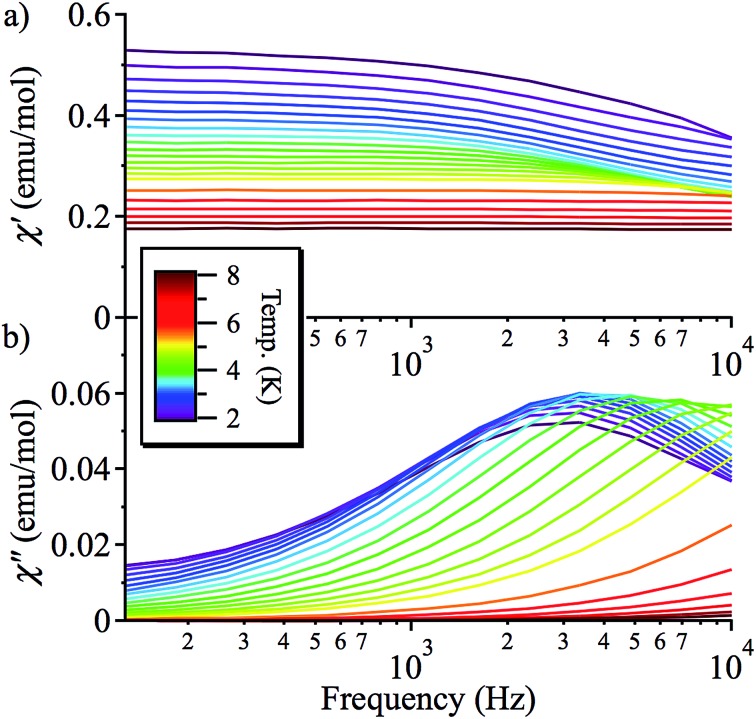
Frequency-dependence of the AC magnetic susceptibility at different temperatures (2–8 K, colour scheme) in a 2000 Oe DC applied magnetic field: (a) in-phase (*χ*′); (b) out-of-phase (*χ*′′) signal.

On the basis of the strong axial nature of **1**, it is interesting to see if it exhibits slow magnetic relaxation, as this behaviour has only very recently been observed for Ni(ii) monometallic complexes and is previously unseen for trigonal bipyramidal Ni(ii).^17,18^ In zero applied DC field, **1** does not display a frequency dependent out-of-phase AC response, due to efficient zero-field quantum tunnelling. However, by using an applied DC field to suppress tunnelling, **1** does display slow magnetic relaxation at low temperature (Fig. 5 and S7†). The data for different applied DC fields are shown in Fig. S7:† as *H*_DC_ increases, the magnetisation of more and more molecules of **1** starts to block, so the *χ*′′ response grows. This is consistent with a very broad distribution of tunnelling rates, as expected because these measurements were performed on a powder sample.

Fitting *χ*′′ to a modified Debye equation yielded the peak positions,^19^ and thus the characteristic relaxation times (*τ*) between 2 and 8 K. The temperature-dependence of these relaxation times is often modelled with an Arrhenius law, which describes thermal relaxation over an energy barrier (Orbach process):

2 *τ* = *τ*_0_ exp(Δ*E*/*kT*)

**Fig. 6:**
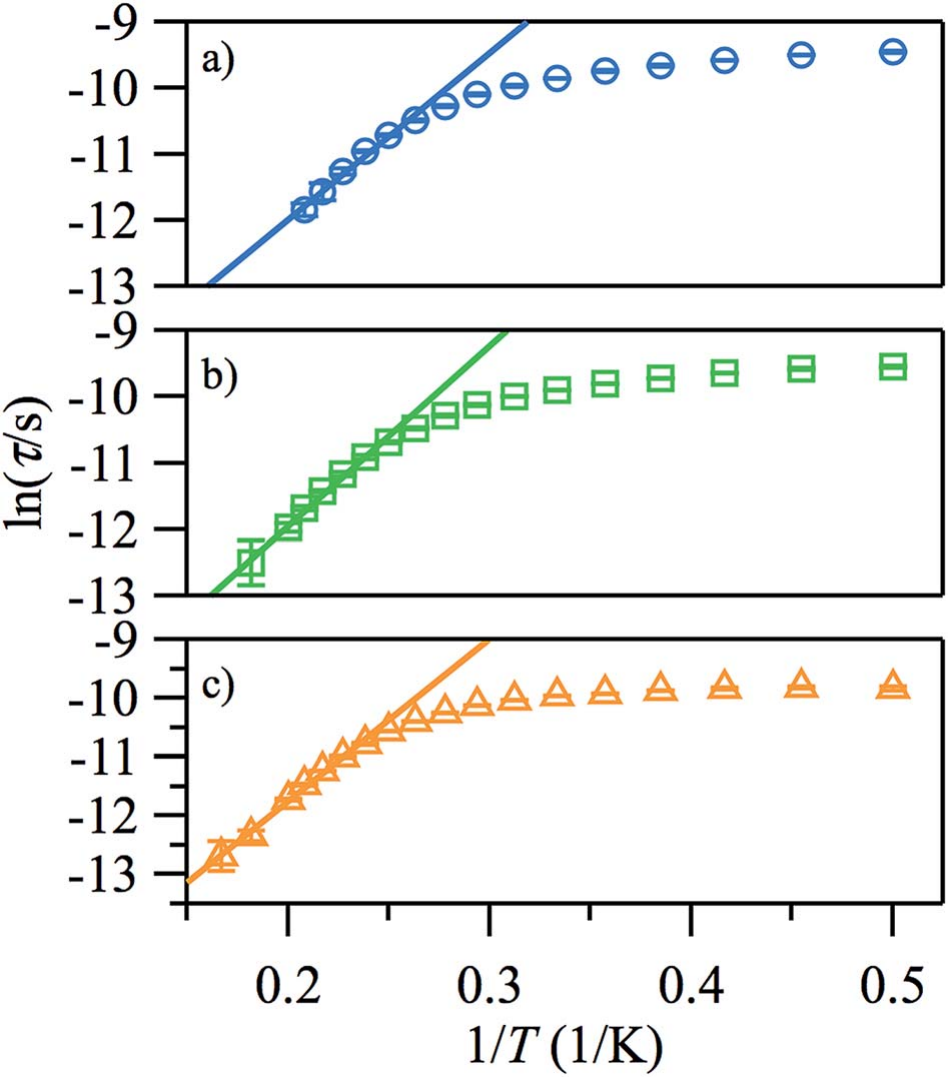
Arrhenius plots of the temperature-dependence of the relaxation times of **1** from *χ*′′ at *H*_DC_ = 500 (a), 1000 Oe (b), 2000 Oe (c).

The plots of ln(*τ*) *vs.* 1/*T* (see Fig. 6), however, are approximately linear only above ∼4.2 K, indicating the importance of other relaxation processes. The quasi-linear region in the ln(*τ*) *vs.* 1/*T* plots can be fitted using eqn (2) to obtain activation energies Δ*E*/*k* of 25.2 (20), 27.1 (10), and 27.8 (9) K for *H*_DC_ = 500, 1000 and 2000 Oe, respectively, with *τ*_0_-values of 4.1 (14) × 10^–8^, 2.8 (6) × 10^–8^, and 3.1 (5) × 10^–8^ s, although it is unlikely that this is Arrhenius behaviour. For monometallic species, especially in applied DC fields, rather than an Orbach process, thermal relaxation can occur *via* Raman or direct spin–phonon processes in addition to the relaxation *via* quantum tunnelling.^20^ Hence, a better way to describe the relaxation is as a combination of different spin–lattice relaxation processes.^21^

3 1/*τ* = *B*_1_/(1 + *B*_2_*H*^2^) + *CT*^*n*^

**Fig. 7:**
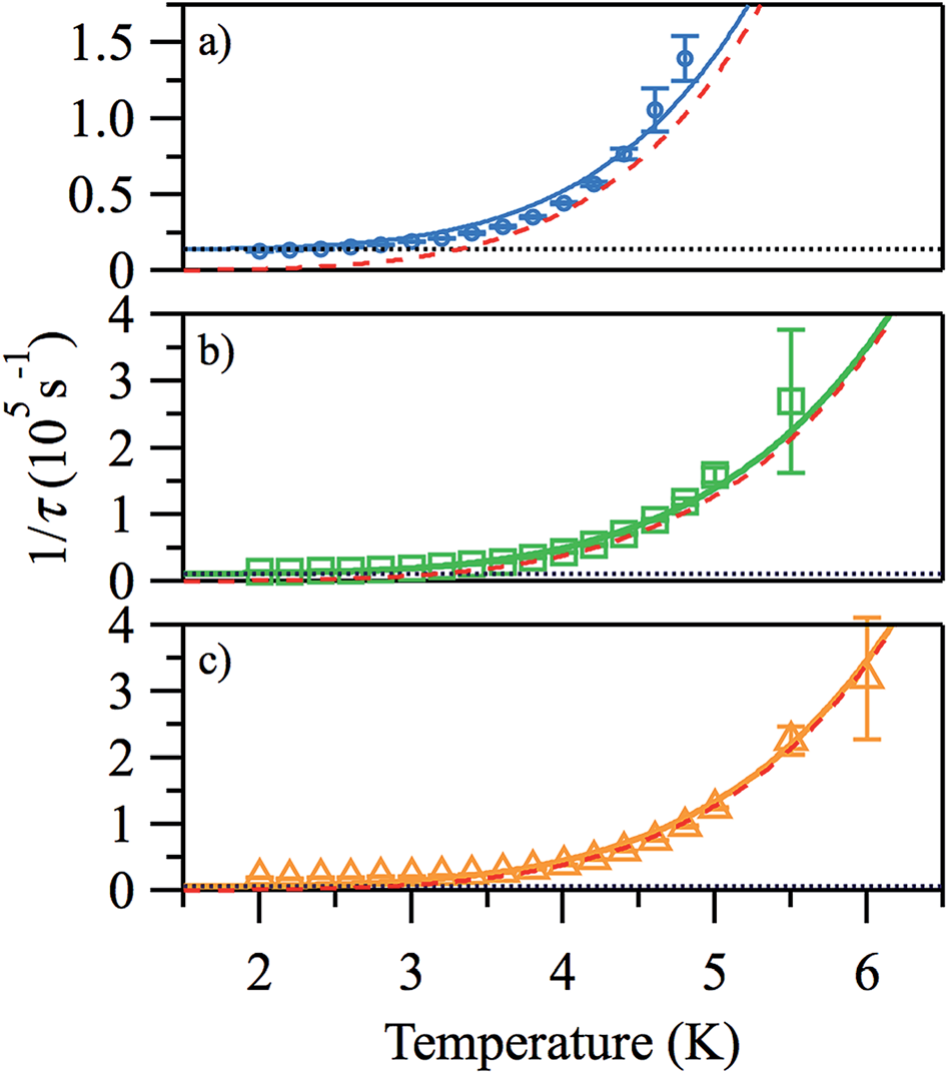
Temperature-dependence of the relaxation rates 1/*τ* from *χ*′′ at *H*_DC_ = 500 (a), 1000 Oe (b), 2000 Oe (c). Open symbols, experimental rates; solid lines, total relaxation rate fits; dotted black lines, tunnelling contribution; dashed red lines, Raman contribution.

Good fits were obtained using eqn (3), where relaxation by a Raman process (1/*τ* ∝ *T* ^*n*^, with *n* = 5.4 (2)) dominates the thermal relaxation in **1**, while quantum tunnelling provides the temperature-independent relaxation at low temperatures (Fig. 7).

### Outlook

All analysis of the data point to a giant magnetic anisotropy, indicating that [Ni(MDABCO)_2_Cl_3_]^+^ is close to an orbital degeneracy and much more so than seen previously.^9^ The axial anisotropy determined from the EPR measurements is much closer to the value calculated for the molecule where 3-fold symmetry is imposed in ref 10. This is interesting and could suggest that small distortions of the TBP structure away from ideal 3-fold symmetry are not as important as the calculations imply, most likely due to the almost unquenched orbital moment in **1**.^20^ Further studies into this relationship are warranted. An analysis of the data that takes into account the orbital degrees of freedom will be explored in future work, which will also include doping **1** into diamagnetic hosts to probe further the spin–lattice relaxation. We have been unable to prepare a Zn analogue of **1** even using our modified synthetic procedure, which leads to four-coordinate {Zn(MDABCO)Cl_3_} complexes. Other interesting synthetic targets include the unknown bromide analogue of **1**, which could help further minimise equatorial distortions and may enhance spin–orbit effects, or [Ni(MDABCO)_2_Cl_3_][X] (where X is a different anion) to try and crystallise [Ni(MDABCO)_2_Cl_3_]^+^ with fewer independent molecules in the unit cell to simplify EPR studies. Single crystal magnetic susceptibility studies and single crystal optical studies should also provide useful information in these types of system.^22,23^

Our experimental observations show that the suppression of Jahn–Teller effects in trigonal bipyramidal Ni(ii) by using rigid, bulky ligands in the axial positions, leads to a potential high anisotropy building block for a new generation of SMMs with improved blocking temperatures. It should be noted that the over-barrier Orbach relaxation mechanism that is important in polynuclear SMMs is not the main relaxation mechanism in many monometallic systems, including **1**.^24^ Hence, the current challenge is to determine how to suppress the unwanted under-barrier relaxation that occurs *via* Raman and direct spin–lattice relaxation processes. The next target is to couple a small number of trigonal bipyramidal Ni(ii) centres, with the addition of an ion with a larger spin along the axial direction. The advantage of such relatively low nuclearity systems will be to remove the spin–lattice and quantum tunnelling relaxation pathways that work effectively for small spin states, but not for larger ones. Clearly, keeping the trigonal environment while maintaining both the symmetry and rigidity of the molecule will be a significant synthetic challenge but nevertheless a rewarding one.
