# Supplementary material for: Pushing the limits of magnetic anisotropy in trigonal bipyramidal Ni(ii)
Source: Chem Sci. 2015 Sep 8;6(12):6823–8. doi: 10.1039/c5sc02854j (PMC5508675; doi:10.1039/c5sc02854j)
Supplement: Supplementary file 1 [file SC-006-C5SC02854J-s001.pdf]

Electronic Supplementary Material (ESI) for Chemical Science.
This journal is © The Royal Society of Chemistry 2015

Pushing the Limits of Magnetic Anisotropy in Trigonal Bipyramidal Ni(II)

Katie E. R. Marriott,[1] Lakshmi Bhaskaran,[2] Claire Wilson,[1] Marisa Medarde,[3] Stefan T. Ochsenbein,[4] Stephen Hill,[2,*] Mark Murrie[1,*]

[1] WestCHEM, School of Chemistry, University of Glasgow, Glasgow, G12 8QQ, UK

[2] Department of Physics and NHMFL, Florida State University, Tallahassee, FL 32310, USA

[3] Laboratory for Developments and Methods, Paul Scherrer Institute, CH-5232 Villigen PSI, Switzerland

[4] Laboratory for Neutron Scattering and Imaging, Paul Scherrer Institute, CH-5232 Villigen PSI, Switzerland

*email: shill@magnet.fsu.edu; mark.murrie@glasgow.ac.uk

Table of contents

1.0 Experimental and synthetic details S3

2.0 Single crystal X-ray diffraction S5

3.0 Powder X-ray diffraction S8

4.0 High-Field EPR S9

5.0 Ac magnetic susceptibility S13

6.0 References S15

1.0 Experimental and Synthetic Details

Materials and Instrumentation

All operations were carried out under aerobic conditions. All reagents and solvents were obtained from commercial suppliers and used without further purification.

Single crystal diffraction data were collected at 100 K on a Bruker APEX-II CCD diffractometer (Mo Kα radiation, λ = 0.71073 Å). The structure was solved using Superflip[1] and refined using full-matrix least-squares refinement on F^2 using SHELXL2014 in OLEX2.[2] All non-hydrogen atoms were refined with anisotropic atomic displacement parameters. All CH_2 hydrogen atoms were placed in geometrically calculated positions and included in the refinement as part of a riding model, Me hydrogen atoms were refined as part of a rigid rotor. All hydrogen atoms were assigned Uiso values at 1.5(Ueq) for the parent carbon atom. Data have been deposited at the Cambridge Structural Database (CCDC number 1059709).

The powder X-ray pattern for **1** was collected on a PANalytical XPert MPD, with Cu Kα1 radiation at ambient temperature for 3 h over a range of 5° < 2θ < 50° using a step size of 0.0167°.

Polycrystalline samples of $[Ni(MDABCO)_2Cl_3]ClO_4$ (**1**) were carefully ground to a powder and encased in eicosane to prevent torquing. Variable temperature dc magnetic measurements were carried using a Quantum Design MPMS-XL SQUID magnetometer in applied dc field of 1000 Oe. Data have been corrected for diamagnetic contributions of the sample holder by measurement and for the sample using Pascal’s constants. AC magnetic susceptibility measurements were carried out using a Quantum Design PPMS at the Laboratory for Developments and Methods, Paul Scherrer Institute, Villigen, Switzerland. The ac data (H_{dc} > 0) have been corrected by subtraction of a very weak signal arising at zero applied dc field (H_{dc} = 0) which shows no shift in the out-of-phase signal with frequency (see Fig S7). This behaviour could arise from a very small impurity phase, although the analytical data and powder X-ray diffraction pattern indicate high sample purity, *vide infra*. However, this could also be an artefact (note that the χ'' signal at H_{dc}=0 is less than 1% of the χ' signal and that the weak χ'' signal increases with increasing frequency and then turns weakly negative above 6 kHz).

High-field EPR measurements were carried out on oriented single-crystal samples in the 50 to 80 GHz range, using a 35 T resistive magnet at the US National High Magnetic Field Laboratory.[3] Low-field measurements were also performed on a single-crystal in the 20 to

100 GHz range, using a 7 T split-pair superconducting magnet.[4] Both setups employed a cavity perturbation technique with *in situ* sample rotation capabilities, while a millimetre-wave vector network analyzer was employed as a microwave source and detector. A commercial Bruker E680 instrument was used for a single crystal EPR measurement at a frequency close to 9.7 GHz. Powder EPR measurements were performed on a pressed pellet of pure ground polycrystalline sample in the 40 to 200 GHz range. The spectrometer used for these measurements relies on quasi-optical light transmission and consists of a tuneable frequency source coupled with a multiplier to provide the final frequency.[5] All the spectra, *i.e.*, single-crystal, powder and X-band were recorded at ~4.2 K.

IR spectra were recorded using a Shimadzu FTIR-8400S spectrophotometer. Micro analysis was carried out on an Exeter CE-440 Elemental Analyser. ^{1}H NMR spectra were recorded using a Bruker AVI 400 MHz Spectrometer. All spectra were recorded at 300 K. Chemical shifts (δ) are stated in parts per million (ppm) and coupling constants (J) in Hertz (Hz). Multiplicities are reported as singlet (s) and triplet (t).

1-Methyl-4-aza-1-azoniabicyclo[2.2.2]octanium iodide ([MDABCO][I]) and [Ni(MDABCO)$_2$Cl$_3$]ClO$_4$ (**1**) were synthesised as described in the literature with slight modification.[6,7,8,9]

Synthesis of 1-Methyl-4-aza-1-azoniabicyclo[2.2.2]octanium iodide ([MDABCO][I])

To a solution of 1,4-diazabicyclo[2.2.2]octane (DABCO) (4.0 g, 35 mmol) in ethyl acetate (70 ml), iodobutane (4.5 ml, 7.2 g, 40 mmol) was added drop-wise over 5 minutes, during which a white precipitate formed. The suspension was stirred at ambient temperature for 2 hours and filtered to give a white solid (8.6 g, 97%) that was washed with ethyl acetate (3 × 20 ml) and dried in a desiccator. ^{1}H NMR (400 MHz, CDCl$_3$) δ ppm 3.19 (t, *J*=7.6 Hz, 6 H, 3 CH$_2$) 3.34 (s, 3 H, CH$_3$) 3.65 (t, *J*=7.6 Hz, 6 H, 3 CH$_2$). Selected IR data: ν (cm^{-1})= 2999m, 1421m, 1350w, 1325w, 1286w, 1055s, 912m, 842s, 685s.

Synthesis of [Ni(MDABCO)$_2$Cl$_3$]ClO$_4$ (1)

To a solution of [MDABCO][I] (0.26 g, 1.0 mmol) and NaClO$_4$ (0.12 g, 1.0 mmol) in MeOH (5ml) a solution NiCl$_2$ (0.26 g, 2.0 mmol) in MeOH (5ml) was added. The solution was stirred at ambient temperature for 5 hours and then filtered. Red block-like single crystals of [Ni(MDABCO)Cl$_3$]ClO$_4$ were formed upon vapour diffusion with diethyl ether, in 19% yield. Selected IR data: ν (cm^{-1}) = 3003m, 1496m, 1359w, 1319w, 1286w, 1080s, 1049s, 1012m, 923m, 852m 845s, 621s. Analysis, calc. (found) for C$_{14}$H$_{30}$Cl$_4$N$_4$NiO$_4$ (**1**): C, 32.40 (32.21), H, 5.83 (5.79), N, 10.79 (10.69).

2.0 Single Crystal X-ray Diffraction

Table S1 Crystal Data and Structure Refinement Parameters

Empirical formula	$C_{14}H_{30}Cl_4N_4NiO_4$
Formula weight	518.93
Temperature / K	100.0 (2)
Crystal system	Orthorhombic
Space group	$Pca2_1$
a / Å	12.3629(16)
b / Å	12.8888(19)
c / Å	13.1011(18)
α / °	90
β / °	90
γ / °	90
Volume / $Å^3$	2087.6(5)
Z	4
ρ_{calc} g/cm^3	1.651
μ / mm^{-1}	1.470
F(000)	1080.0
Crystal size / mm^3	0.45 × 0.4 × 0.24
Radiation	MoKα (λ = 0.71073)
2Θ range for data collection / °	3.16 to 54.968
Index ranges	$-15 \leq h \leq 14$, $-15 \leq k \leq 16$, $-16 \leq l \leq 10$
Reflections collected	12167
Independent reflections	3646 [R_{int} = 0.0781, R_{sigma} = 0.0795]
Data / restraints / parameters	3646 / 1 / 247
Goodness-of-fit on F^2	1.067
Final R indexes [I > = 2σ (I)]	R_1 = 0.0438, wR_2 = 0.0949
Final R indexes [all data]	R_1 = 0.0543, wR_2 = 0.1000
Largest diff. peak/hole / e $Å^{-3}$	0.47/-0.70
Flack parameter	0.16(3)

Table S1 - Selected bond lengths, contacts (Å) and angles (°) for 1

Ni – N1	2.191(5)
Ni – N3	2.196(5)
Ni – Cl1	2.310(17)
Ni – Cl2	2.318(18)
Ni – Cl3	2.315(15)
Ni ··· Ni	8.902(1) - 9.717(1)
N1-Ni-Cl1	88.58(14)
N1-Ni-Cl2	91.41(14)
N1-Ni-Cl3	90.26(12)
N3-Ni-Cl1	88.66(13)
N3-Ni-Cl2	91.62(13)
N3-Ni-Cl3	89.59(12)
Cl1-Ni-Cl2	117.03(7)
Cl2-Ni-Cl3	119.73(7)
Cl1-Ni-Cl3	123.24(7)
N1-Ni-N3	176.59(19)

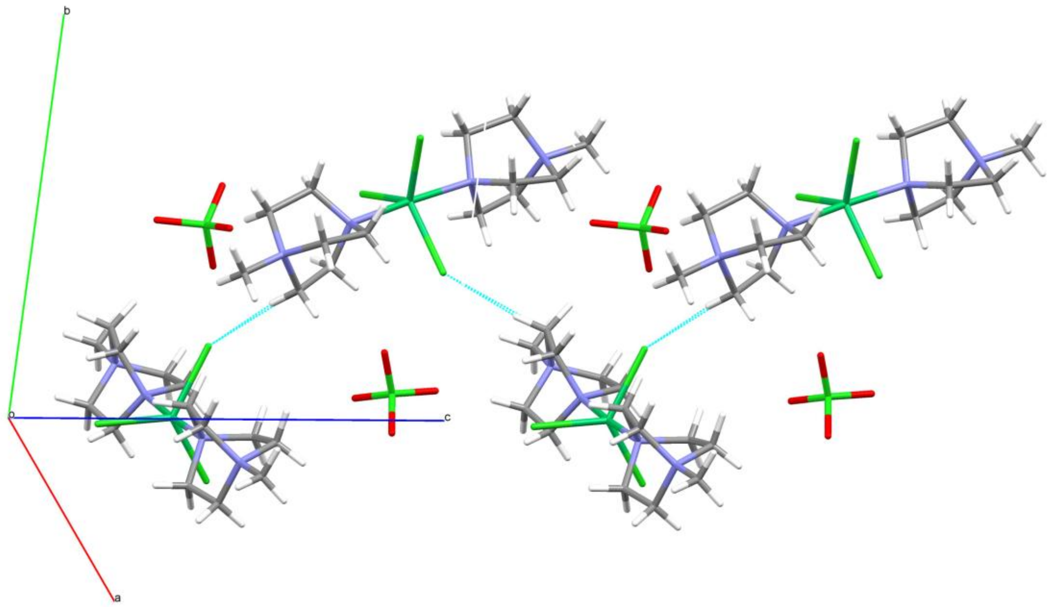

Figure S1 Examination of the H…Cl contacts in the structure shows one (C13-H13B...Cl1[a] H…Cl 2.47Å, C…Cl 3.430(6) Å, <CHCl 172.7 ° a) 1-X,1-Y,-1/2+Z) which is close to linear at the hydrogen atom and falls in the 'short' category proposed in Ref. [10]. However, the Ni…Ni separation here is 9.270(2) Å. Hydrogen bonds are shown as cyan lines. C, grey; Cl, pale green; H, white; N, blue; Ni, green.

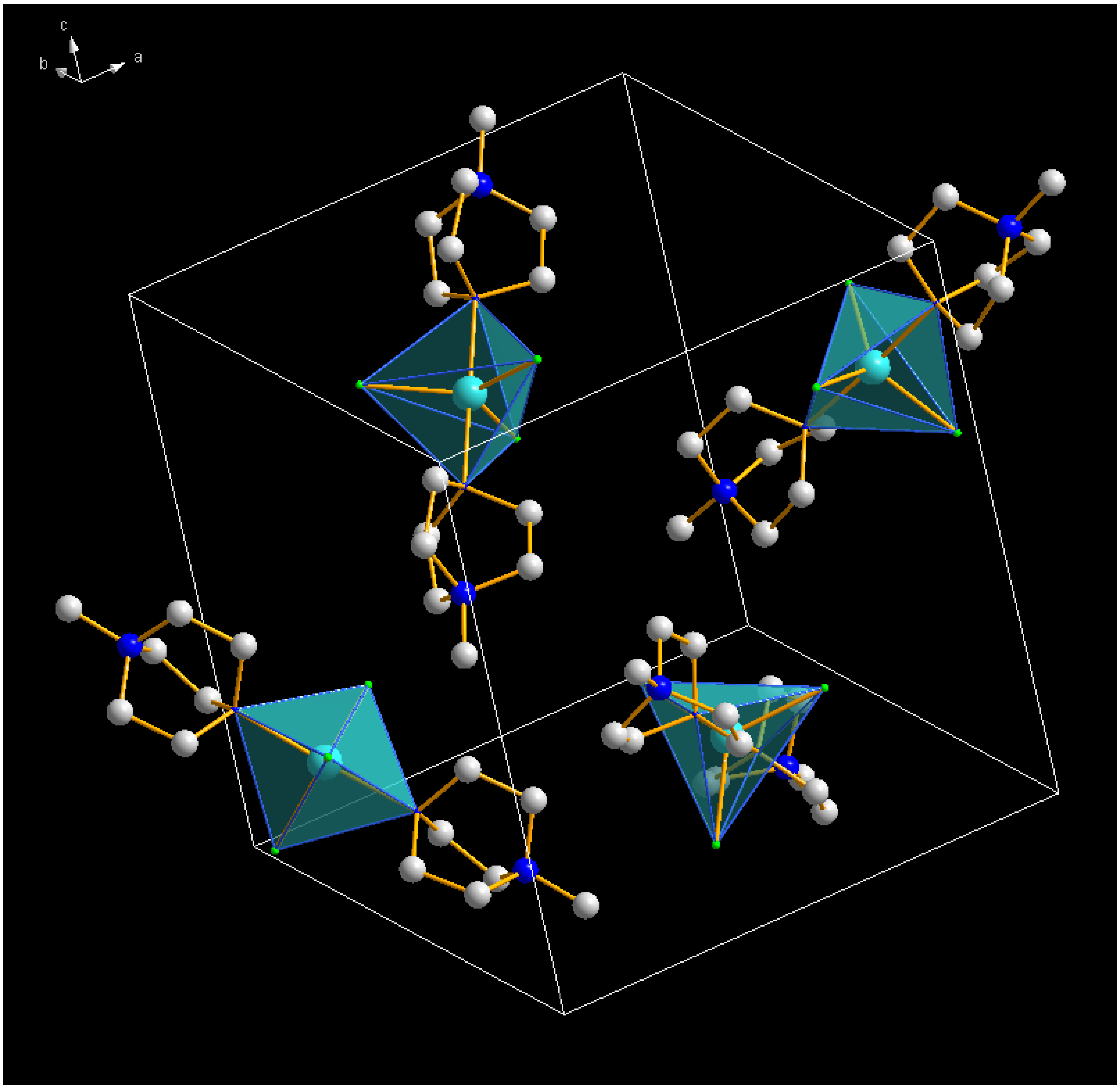

Figure S2 Crystal packing highlighting the four differently oriented molecules within the unit cell. C, grey; Cl, green; N, blue; Ni, cyan. H atoms and ClO_4^- anions omitted for clarity.

3.0 Powder X-ray Diffraction

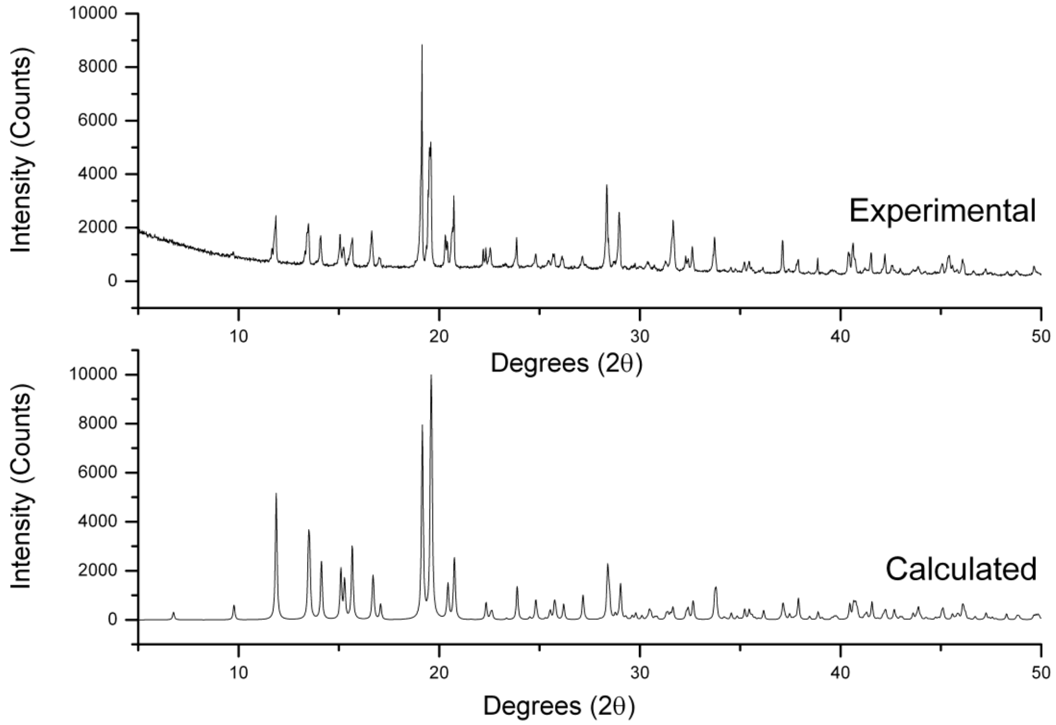

Figure S3 Powder X-ray diffraction pattern for a crushed sample of **1** (upper) and the calculated pattern from the single crystal data (lower), both at ambient temperature.

4.0 High-Field EPR

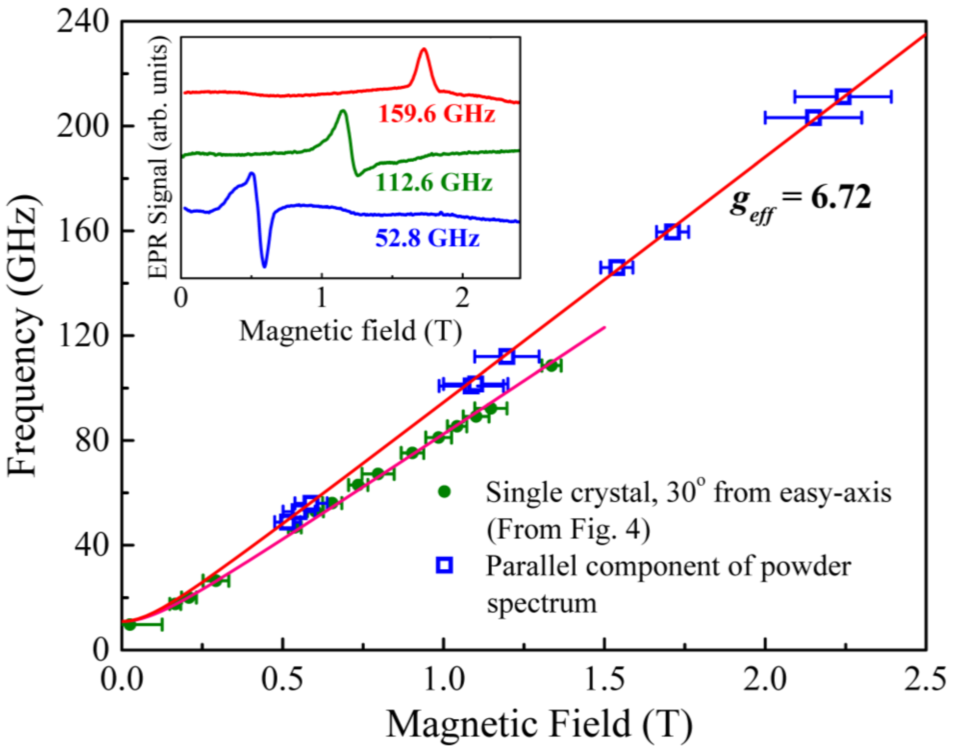

Figure S4 Comparison between the frequency dependence of the peak positions determined from powder and single crystal measurements. The inset displays several representative powder spectra, which were recorded in field derivative mode (*dI*/*dB*, where *I* is the EPR absorption intensity), with the frequencies indicated. The strong signal in the derivative corresponds to the onset of absorption among the randomly oriented crystallites within the powder, corresponding to the *z*-component of the spectrum. Multiple peaks were observed for the single-crystal, corresponding to the differently orientated species within the unit cell of **1**; the green data points (solid circles) in the main panel of the figure correspond to the signals from the species that had its easy-axis closest to alignment with the applied field. From the single-crystal measurements performed at the lowest frequency (9.7 GHz), we ascertain that the zero-field gap between the lowest-lying pair of singlets (T_x and T_y) must be at or below 11 GHz, giving an upper bound on the *E* value associated with the spin-only description of **1** of ~0.18 cm^{-1}. Fits to the data yield effective *g*-values: the powder data yield an effective value corresponding to twice the actual value of g_z = 3.36 within the spin-only description; the crystal data give a somewhat lower *g*-value due to the fact that the field was not perfectly aligned with the easy-axis of any of the four species within the unit cell. In other words, the powder data were used to constrain g_z; meanwhile the single-crystal data, that could be performed to much lower frequencies, were used to set an upper bound on *E*.

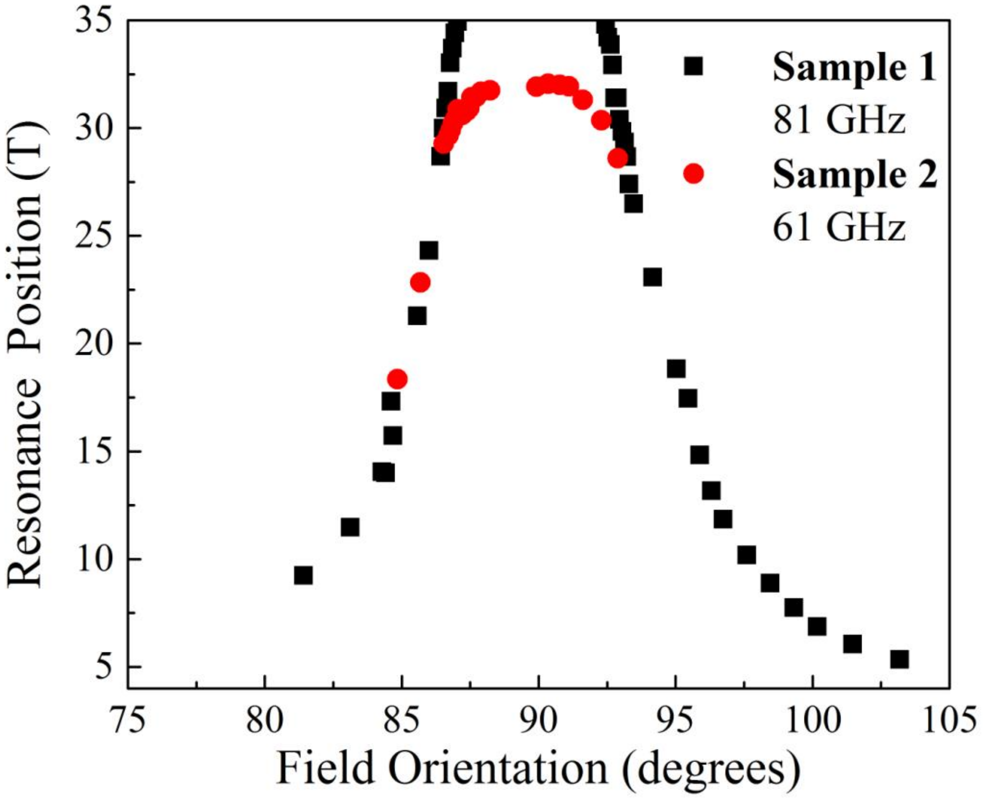

Figure S5 Representative angle-dependent high-field EPR data for two separate samples, illustrating the very strong magnetic anisotropy of compound **1**. The temperature was 4.2 K and the frequencies are given in the figure. The resonance positions were determined from field swept measurements, which were then repeated at fine angle steps in order to accurately locate the hard plane of the sample (the maximum in the angle-dependence, set to 90 degrees in the figure). Note that the measurements were conducted for each sample for an arbitrary plane of field rotation: neither the inclination of this plane, nor its intersection within the hard plane was known. Therefore, one cannot directly compare the angle-dependence obtained for the two samples. The data were used solely to locate the sample hard plane. Frequency-dependent measurements were then performed and analysed at this orientation (See Figure S6).

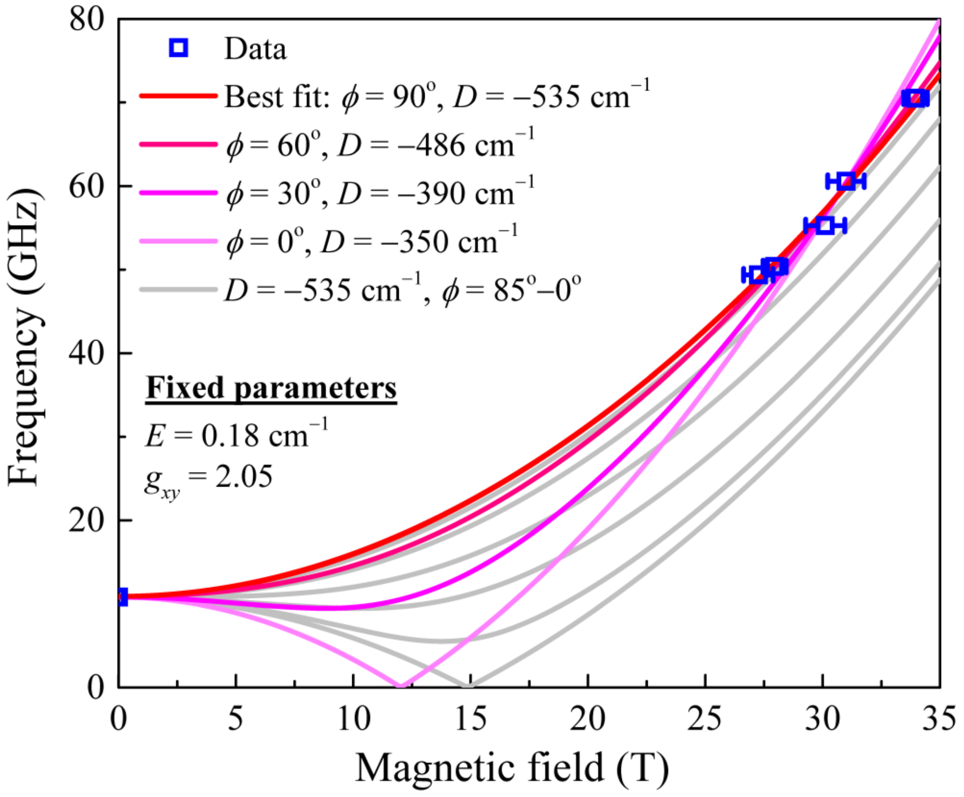

Figure S6 Various simulations and fits to the high-field EPR spectra obtained with the magnetic field in the hard-plane of one of the four species within the unit cell; all fits were performed using a spin-only model. Because the orientation of the applied field within the hard plane was not known, the gray curves illustrate the effect of the *xy* anisotropy caused by a finite rhombic *E* term. Note that all curves are highly constrained at zero field by the value of Δ_E (0.36 cm^{-1}) determined via the low field measurements in Figure S4. However, the high-field spectrometer does not permit measurements below 50 GHz. The curves were generated for a fixed value of D = –535 cm^{-1}, for different field orientations, ϕ, within the hard plane; the ϕ = 90° orientation corresponds to the best fit. Because the plane of rotation is unknown, one can re-fit the data for different orientations, ϕ, whilst allowing D to vary (keeping fixed E = 0.18 cm^{-1} and g_{xy} = 2.05). The red and pink curves correspond to such fits. As can be seen, the ϕ = 90° curve has precisely the right slope to intersect the high-field data, whereas the shapes of the curves closer to ϕ = 0° do not sit well on the data. Obviously the *g* values can be adjusted to correct the slopes of each curve. However, this procedure requires further adjustments to *D* (see discussion of the *g*-value uncertainty below), eventually resulting in completely unphysical *g* values (<0.4) in order to achieve agreement with the experimental data. On this basis, we estimate a very conservative lower bound for the absolute value of *D* of 400 cm^{-1} due to the lack of knowledge of the field rotation plane.

Uncertainty in the *g*-values

The remaining source of uncertainty in the spin only parameterisation of the EPR data concerns the strong inter-dependence between D and the values of g_x and g_y. Because of the strong uniaxiality of **1**, we set $g_x = g_y = g_{xy}$. However, fits to the EPR data do not converge if both D and g_{xy} are allowed to vary simultaneously. For this reason, g_{xy} was set to 2.05 on the basis of fits to the magnetic data. Additional fits to the high-field EPR data in Fig. S6 (not shown) were performed with different values of g_{xy} in order to determine what effect this has on D. It is found that if a multiplier, α, is applied to g_{xy}, then the best fit D value is scaled by α^2. Fit curves generated with the scaled and unscaled parameters sit right on top of each other. This finding is relatively straightforward to understand on the basis of a perturbative treatment of the Zeeman interaction. In other words, if $g_{xy} = 2.20$, then the best fit D value is $-535 \times (2.20/2.05)^2$ cm^{-1} = -615 cm^{-1}, while a g_{xy} value of 2.00 gives $D = -510$ cm^{-1}; we note that a $g_{xy} < 2.00$ is unphysical.

5.0 Ac Magnetic Susceptibility

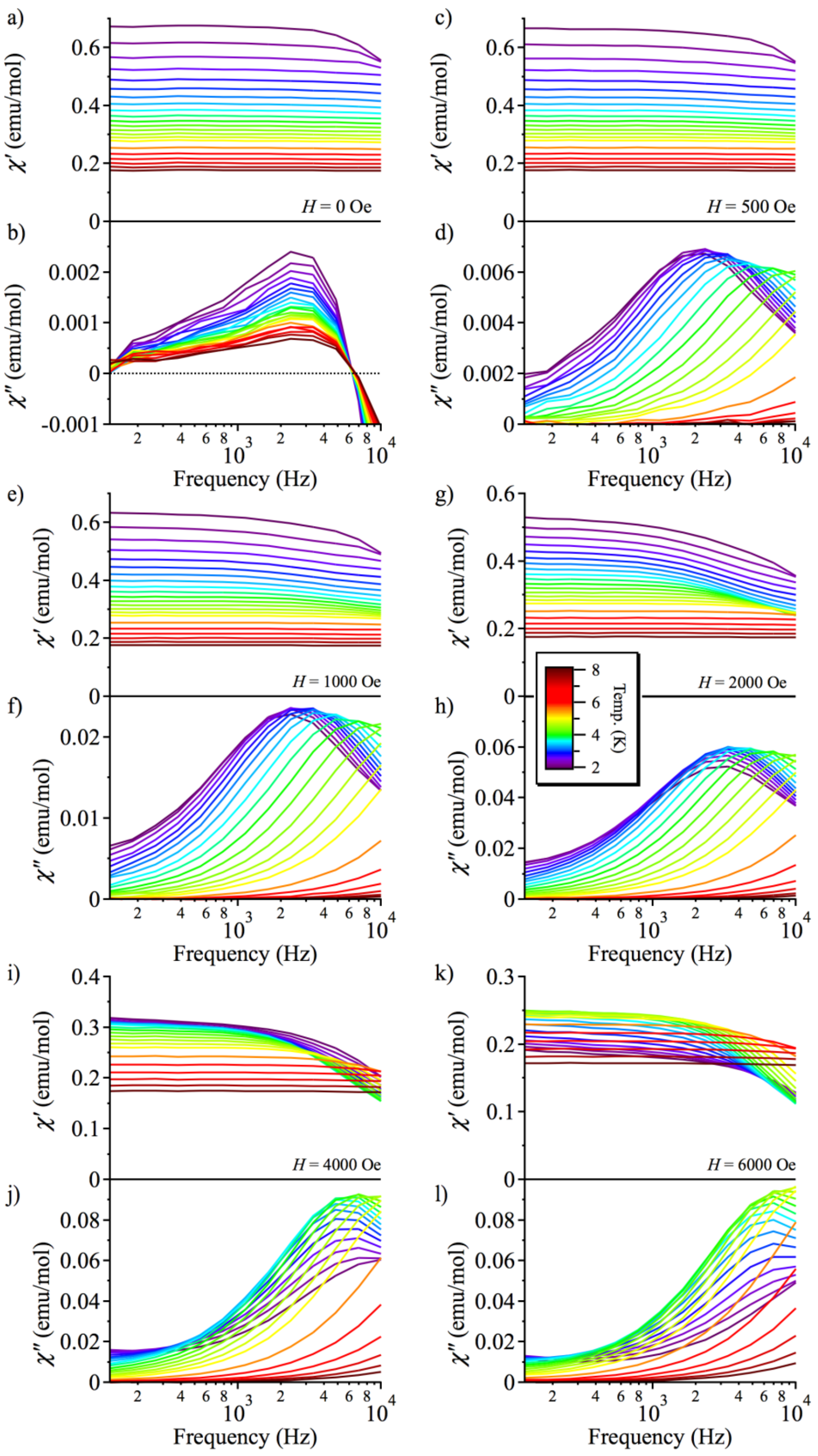

Figure S7 Frequency-dependence of the *ac* magnetic susceptibility at different temperatures (2 – 8 K, colour scheme) in different *dc* magnetic fields H_{dc}. The ac data ($H_{dc} > 0$) have been corrected by subtraction of the weak signal arising at zero applied dc field ($H_{dc} = 0$) (for an explanation see section 1.0).

The Argand plots of the ac magnetic susceptibility data show temperature-dependent behaviour (see Fig. S8). The deviations from nice semi-circles, especially pronounced at H_{dc} = 500 and 1000 Oe, suggest a variety of processes are contributing to the relaxation.

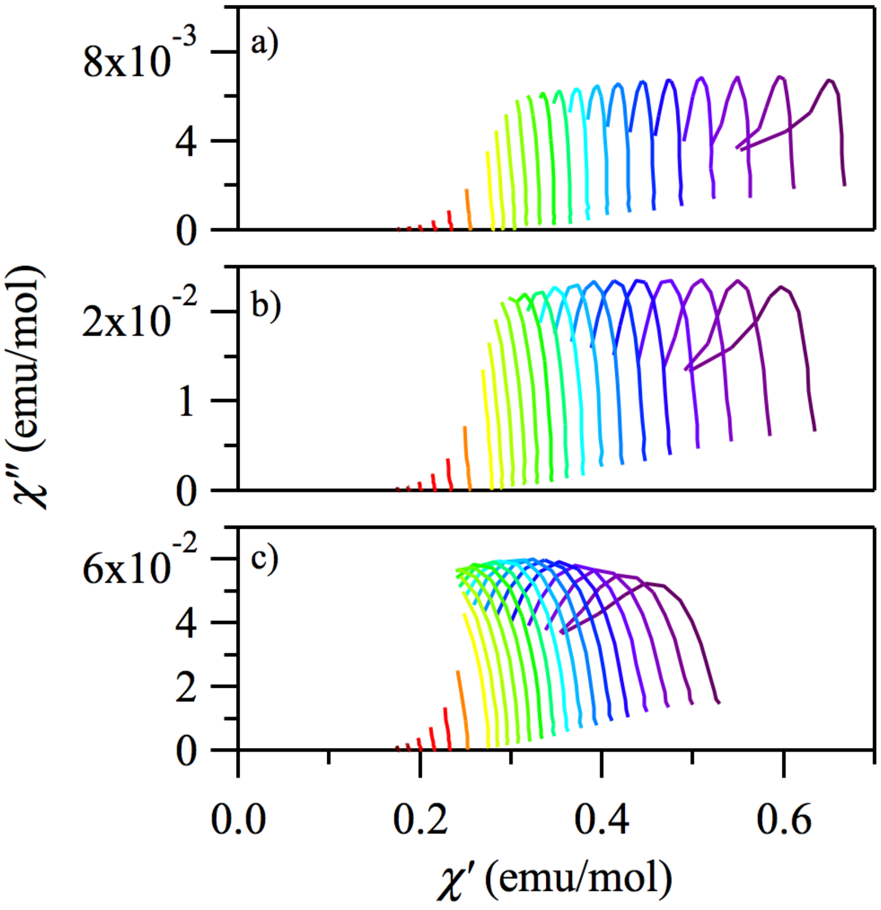

Figure S8 Argand plots of the *ac* magnetic susceptibility of **1** at H_{dc} = 500 Oe (a), 1000 Oe (b), and 2000 Oe (c).

6.0 References

1. L. Palatinus and G. Chapuis, *J. Appl. Cryst.* 2007, **40**, 786–790.

2. O. V. Dolomanov, L. J. Bourhis, R. J. Gildea, J. A. K. Howard and H. Puschmann, *J. Appl. Cryst.* 2009, **42**, 339–341.

3. M. Mola, S. Hill, P. Goy and M. Gross, *Rev. Sci. Inst.* 2000, **71**, 186.

4. S. Takahashi and S. Hill, *Rev. Sci. Inst.* 2005, **76**, 023114.

5. A. K. Hassan, L. A. Pardi, J. Krzystek, A. Sienkiewicz, P. Goy, M. Rohrer and L.-C. Brunel, *J. Magn. Reson.* 2000, **142**, 300–312.

6. J. Rozell and J. S. Wood, *Inorg. Chem.* 1977, **16**, 1827–1833.

7. L. M. Vallarino, V. L. Goedken and J. V Quagliano, *Inorg. Chem.* 1972, **11**, 1466–1469.

8. D. V. Konarev, S. S. Khasanov, A. Otsuka, G. Saito and R. N. Lyubovskaya, *Inorg. Chem*. 2007, **46**, 2261–2271.

9. J.-Y. Kazock, M. Taggougui, B. Carré, P. Willmann and D. Lemordant, *Synthesis (Stuttg).* 2007, **24**, 3776–3778.

10. G. Aullón, D. Bellamy, A. G. Orpen, L. Brammer and E. A. Bruton, E. A. *Chem. Commun.* 1998, **6** 653–654.
